# APVCPC: An Adaptive Predicted Value Computation and Pixel Classification Framework for Reversible Data Hiding in Encrypted Images

**DOI:** 10.3390/s26051636

**Published:** 2026-03-05

**Authors:** Yaomin Wang, Wenguang He, Gangqiang Xiong, Yuyun Chen

**Affiliations:** School of Biomedical Engineering, Guangdong Medical University, Dongguan 523808, China; wym1213@gdmu.edu.cn (Y.W.); hewenguang@gdmu.edu.cn (W.H.); xgq@gdmu.edu.cn (G.X.)

**Keywords:** multimedia security, image encryption, data hiding, high capacity

## Abstract

With the proliferation of Internet of Things (IoT) deployments and mobile sensing systems, reversible data hiding in encrypted images (RDHEI) has emerged as a cornerstone technology for secure cloud-based sensor data management. RDHEI ensures data confidentiality while enabling bit-to-bit restoration of original visual assets. However, conventional RDHEI methods often struggle to optimize the trade-off between high embedding capacity (EC) and the fidelity requirements of sensor-acquired content. This paper proposes an advanced RDHEI framework based on Adaptive Predicted Value Computation and Pixel Classification (APVCPC). The core contribution is a context-aware prediction engine that adaptively selects optimal estimation functions based on local texture complexity, significantly enhancing prediction accuracy in heterogeneous image regions. Subsequently, a content-driven pixel classification paradigm categorizes pixels into loadable (Lpxls) and non-loadable (NLpxls) sets using a dynamic threshold, maximizing the utilization of spatial redundancy. The proposed scheme further supports separable data extraction and image decryption, providing flexible access control for diverse user privileges in secure sensing scenarios. Experimental results on standard benchmarks and the BOW-2 database demonstrate that APVCPC achieves a superior average embedding rate exceeding 2.0 bpp and ensures perfect reversibility, significantly outperforming state-of-the-art techniques in terms of both capacity and security.

## 1. Introduction

Unprecedented volumes of visual sensing data have been generated by the extensive deployment of Internet of Things (IoT) sensors and Wireless Multimedia Sensor Networks (WMSNs), where effective data collection is vital for environment perception [1,2]. These data streams are increasingly offloaded to cloud infrastructures for storage and analytical processing [3]. In such sensor-rich ecosystems, maintaining data privacy and content authenticity is paramount when handling sensitive telemetry or proprietary visual assets. Consequently, reversible data hiding (RDH) has emerged as a key technology for integrating supplementary information—such as sensor metadata or authentication signatures—into digital imagery while ensuring bit-to-bit restoration of the original sensing content [4].

While the RDH field has evolved through difference expansion and histogram modification [5], the escalating risk of cyber-threats necessitates robust encryption prior to cloud transmission. Specialized encryption techniques for sensor nodes now provide multilevel privacy protection but transform images into high-entropy, noise-like representations [6]. This effectively neutralizes the spatial redundancy that conventional RDH schemes rely upon. To address this, reversible data hiding in encrypted images (RDHEI) has become a specialized paradigm designed to reconcile the competing demands of cryptographic security and embedding capacity in secure cloud-based sensor management systems.

Substantial progress has been achieved in RDHEI research. The existing methods can be broadly classified into two paradigms according to the operational sequence of encryption and embedding: Vacating Room After Encryption (VRAE) and Reserving Room Before Encryption (RRBE) [7,8]. VRAE embeds data after image encryption, whereas RRBE pre-allocates embedding regions prior to encryption. A seminal VRAE framework proposed in [7] operates through block-based processing. The host image is partitioned into non-overlapping blocks, where each block embeds one data bit via selective inversion of three least significant bits (LSBs) in half of its pixels. Data extraction relies on a complexity-aware fluctuation function, necessitating sufficiently large block sizes to ensure error-free reversibility. Inadequate block dimensions risk extraction errors and irreversible image degradation. Hong [8] improved this approach using a “side match” technique to reduce extraction errors for fixed block sizes. Subsequent refinements [9,10,11] optimized the fluctuation function, yet these enhancements retained limited embedding capacity despite achieving reversibility. To address capacity constraints, Wu et al. [12] introduced a chessboard-like pixel classification into “qualified” and “forbidden” sets. Qualified pixels carry payloads via multi-bit LSB flipping (≥4 bits), while forbidden pixels facilitate lossless prediction. This innovation significantly boosts embedding capacity while maintaining reversibility through structured pixel utilization.

All the aforementioned methods are categorized as joint RDHEI since data extraction has to be carried out subsequent to image decryption. Zhang pioneered the first separable RDHEI approach by introducing the idea of compressing least significant bits (LSBs) [13]. Following this, lossless compression techniques have also been incorporated into other related methods [14]. Some RDHEI schemes [15,16] achieve the liberation of embedding capacity by utilizing low density parity check code (LDPC) as the compression mechanism. The work in [17] generates space for data hiding by leveraging Hamming distance. In [18], researchers designed a universal RDHEI by exploiting Golomb–Rice codewords. Several studies [19,20,21] have also developed separable RDHEI methods specifically for JPEG images. Additionally, regarding certain VRAE-based methodologies referenced in [22,23,24], the extraction of confidential data can be performed either before or after image decryption.

The limitation of VRAE methods stems from their limited embedding capacity, which arises due to the disrupted correlation between adjacent pixels. In contrast, RRBE methods offer greater capacity, thus drawing significant research attention. The inaugural RRBE method, presented in [25], divides the cover image into two separate segments: the complex segment and the smooth segment. After that, several least significant bits (LSBs) from pixels in the complex segment were embedded into pixels in the smooth segment to create space for data hiding. Subsequently, to protect the image owner’s privacy, the reconstructed image was encrypted. The encrypted result was then transmitted to a remote server via an insecure channel. At the remote server, confidential data were embedded into the reserved space. In [26], Nguyen et al. further developed the idea of partitioning the original image in a chessboard-like pattern. Prior to image encryption, all pixels in the white set were divided into two parts: the smooth part and the complex part. Pixels in the smooth part were then used for space reservation, while the values of white set pixels were predicted using pixels from the black set. In [27], Zhang et al. proposed an estimation error technique, where most pixels are used to estimate the remaining small number of pixels. In [28], confidential data are embedded into the original image by replacing its lower bit-planes, with the replaced bit-planes transmitted through the higher bit-planes. In [29], researchers utilized the homomorphic and probabilistic properties of the Paillier cryptosystem, randomly selecting pixels for space reservation. The work proposed in [30] adopts patch-level sparse representation, achieving an embedding rate (ER) exceeding 0.5 bpp (bits per pixel). In [31], Malik et al. suggested preprocessing the cover image through prediction-error estimation, resulting in an ER of up to 0.75 bpp. Using reversible contrast mapping, the method propoed in [32] achieved an ER approaching 1 bpp. Given that the most significant bit (MSB) plane has the strongest correlation, Puteaux et al. proposed two MSB prediction-based methods in [33]. While these methods can also reach an ER of 1 bpp, they do not always guarantee reversibility. In [34], the MSB is more effectively utilized to develop an efficient RDHEI method with an ER exceeding 1 bpp. In [35], Wang et al. proposed a block-based adaptive MSB encoding scheme, which can achieve an ER of more than 1 bpp. By leveraging the Laplacian-like distribution characteristic of prediction errors and adaptively selecting between “L”-shaped block embedding and improved binary-block embedding, the method proposed in [36] is capable of achieving an ER higher than 1.5 bpp. Another RDHEI approach, which employs hybrid coding (combining entropy coding and hierarchical coding) to construct the embedding space, is proposed in [37]; this approach can achieve a high ER exceeding 1.5 bpp. Furthermore, a subset of researchers has attempted to introduce machine learning techniques into the field of RDHEI. For instance, Panchikki et al. established an ensemble model in [38], which fuses three distinct machine learning models: Support Vector Machine (SVM), Convolutional Neural Network (CNN), and k-Nearest Neighbor (KNN). Incorporating a voting mechanism, this model enables the extraction of embedded data and the restoration of the original image. As a preliminary exploration of machine learning applications in RDHEI, their proposed algorithm can only achieve an embedding capacity of 0.0625 bpp. Nevertheless, this approach holds considerable promise for addressing the robustness challenges confronting the RDHEI field and represents an extremely valuable research exploration. In summary, existing methods indicate that there is still room for improvement in high-capacity RDHEI, and MSB prediction deserves in-depth exploration.

A novel RDHEI method founded on predicted value adaptive computation (APVCPC) is introduced in this paper. Initially, the pixels in the original image is divided into two categories: candidate pixels and prediction pixels. Next, all candidate pixels are further classified into two types: loadable pixels (Lpxls), which are used to carry confidential data, and non-loadable pixels (NLpxls), which need to be excluded from the data-carrying process. To increase the proportion of loadable pixels—and thereby enhance the overall embedding capacity—a new pixel classification approach based on APVCPC is proposed in this work. The proposed method fabricates the embedding capacity by evacuating several MSBs of the Lpxls to further increase the embedding capacity. Here, the number of substituted MSBs is determined by a pre-defined threshold. Moreover, within the proposed method, the independent accomplishment of image recovery and data extraction is achieved. Upon the acquisition of decryption and data-hiding keys, receivers are thereby empowered to access heterogeneous information. Precisely, those receivers furnished with the data-hiding key possess the capacity to extract the confidential data, while those equipped with the decryption key are in a position to retrieve the original image. In the scenario where both keys are available, the receiver is capable of attaining both the image and the confidential data, thus ensuring a stratified access paradigm and upholding security integrity.

The principal contributions of this paper, which collectively address the longstanding trade-off between embedding capacity and reversibility in RDHEI, are articulated as follows:

- **A Context-Adaptive Prediction Engine for Enhanced Spatial Redundancy Exploitation.** This mechanism breaks through the limitations of static prediction and lay a highly reliable theoretical foundation for subsequent pixel classification. Specifically, we propose a novel prediction paradigm that abandons the one-size-fits-all fixed prediction function and designs a suite of prediction rules tailored to different local complexity contexts. Through an intelligent decision mechanism, it analyzes the statistical distribution (concentration) of each pixel’s neighborhood and dynamically selects the optimal prediction rule for it. This context-sensitive approach can significantly improve prediction accuracy, especially in heterogeneous image regions. It not only achieves a theoretical breakthrough over fixed prediction schemes but, more importantly, builds a more reliable foundation for the subsequent core step of pixel classification, fully exploiting the value of image spatial redundancy.
- **A Prediction-Driven Pixel Classification Paradigm Shifting from Geometry to Content.** This contribution realizes the transformation of pixel classification from geometry-oriented to content-oriented, which is the core driver for improving the algorithm’s embedding capacity. We completely depart from the traditional fixed-pattern (e.g., chessboard) pixel classification approach and propose a dynamic classification method based on the coherence between a pixel’s original value and its adaptively calculated predicted value at the most significant bit (MSB) level, categorizing pixels into loadable pixels (Lpxls) and non-loadable pixels (NLpxls). This content-aware classification can accurately identify pixels whose MSBs are reliably predictable and thus safely vacatable, maximizing the proportion of Lpxls. This strategic shift from geometric to semantic classification is the primary reason for the significant improvement in the embedding capacity of our method.
- **A Holistic Framework Integrating Adaptive MSB Substitution with Guaranteed Separable Reversibility.** This framework integrates the advantages of the preceding modules to achieve efficient embedding, lossless recovery, and flexible access control, enhancing the security and practicality of the algorithm. We design a high-efficiency integrated embedding framework that synergistically combines the advantages of the above two modules: it locks in the optimal carrier pixels (Lpxls) through adaptive classification and strategically vacates their multiple MSB bits to build a stable embedding space. Meanwhile, it ensures the perfect recovery of the original image through a losslessly compressed location map, achieving reversibility. More importantly, the framework is designed from the ground up to support genuine separable operations, adapting to flexible access control in cloud scenarios: data extraction and image recovery are independent processes, enabling users with different keys to access only the information they are authorized for, thereby improving both the security and practical application value of the algorithm.

The rest parts of the paper is constructed as follows: Section 2 provides a detailed description of the proposed method. In Section 3, the experimental results and analysis are presented, while Section 4 concludes with a summary of this paper.

## 2. Proposed Method

**Figure 1:**
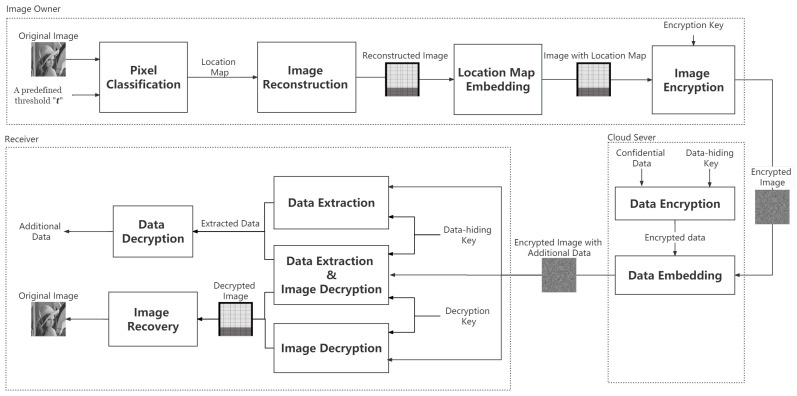
Execution flowchart of the proposed method.

This section elaborates on the details of the proposed method, focusing on four key aspects: (1) details of APVCPC, (2) image encryption, (3) data hiding, and (4) data extraction and image recovery. Figure 1 depicts a schematic diagram of the proposed method. It can be seen that three key entities, namely, the image owner, the cloud server, and the receiver, participate in the execution of the proposed method. Initially, all pixels of original image are classified into two categories: Lpxls and NLpxls. Subsequently, a location map is acquired to guide image reconstruction. After undergoing lossless compression, the location map is smoothly merged into the reconstructed image. Subsequently, the encryption key is applied to conduct the encryption operation on the reconstructed image in tandem with the location map. Upon completion of this encryption process, the encrypted image is then dispatched and transmitted to the cloud server as the final step on the image owner side. Within the cloud server environment, the encryption of the specific confidential data is effected by means of a data-hiding key. Thereafter, the substitution of several MSBs of the Lpxls is enacted so as to embed the encrypted confidential data within the encrypted image. At the receiver end, different receivers with varying access rights to the secret keys may obtain different outcomes.

### 2.1. Details of APVCPC

**Figure 2:**
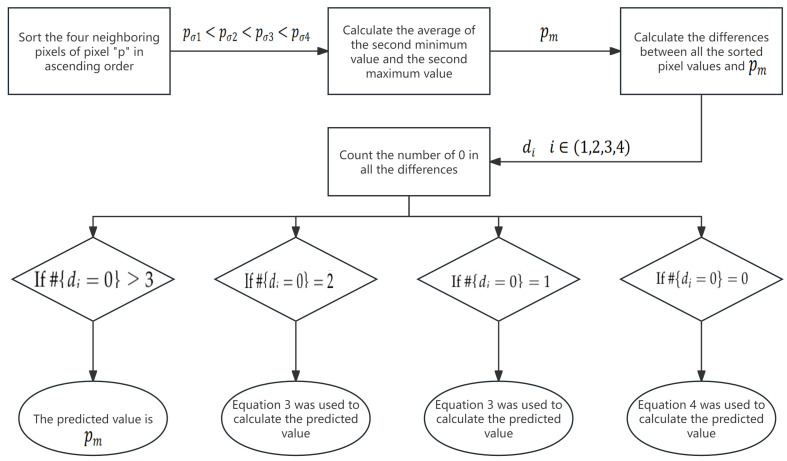
Flow of the predicted value $p_{e}$ calculation.

**Figure 3:**
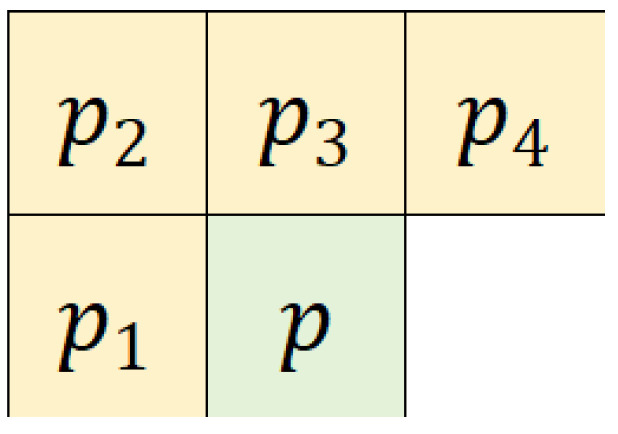
Demonstration of target pixel and its neighboring pixels.

This section provides a comprehensive overview of the APVCPC system, detailing its components, functionality, and operational principles. Table 1 summarizes the symbols employed throughout this paper along with their respective definitions, while Table 2 outlines the specific functions utilized in this paper and their corresponding roles. To simplify the analytical process, the cover image is assumed to have a size of m×n. In APVCPC, the adaptive computation of predicted value is first performed. Initially, pixels in the original image are divided into two sets: the prediction set and the candidate set. The prediction set is composed of the pixels located in the first row, the first column, and the last column, whereas the remaining pixels are allocated to the candidate set. For each pixel within the candidate set, its predicted value $p_{e}$ is initially computed. Figure 2 illustrates the specific procedural steps involved in the calculation of predicted value. The detailed process for calculating $p_{e}$ is described in the following. As manifested in Figure 3, the prediction pixels are chosen from the four neighboring pixels of the candidate pixel *P*, labeled as $p_{1}$, $p_{2}$, $p_{3}$, and $p_{4}$. They undergo a sorting process, yielding the result that $p_{\sigma (1)}\leq p_{\sigma (2)}\leq p_{\sigma (3)}\leq p_{\sigma (4)}$.

Then, their concentration is described as

(1) $$d_{i}=\left|p_{\sigma (i)}-p_{m}\right|\;i=1,2,3,4,$$

where $p_{m}=round\left(\frac{p_{\sigma (2)}+p_{\sigma (3)}}{2}\right)$. As shown in Table 1, the symbol # denotes the cardinality of a set, and the predicted value is determined by the number of elements for which $d_{i}=0$.

Case 1: If $\#\{d_{i}=0\}\geq 3$, the predicted value directly adopts the median reference

(2) $$p_{e}=p_{m}.$$

Case 2: If $\#\{d_{i}=0\}=2$, neither $p_{\sigma (1)}$ nor $p_{\sigma (4)}$ is equal to $p_{m}$. $p_{e}$ is computed as

(3) $$p_{e}=\left\{\begin{array}{cc} p_{m}\;\times \;0.5\;+\;\frac{\sum_{i\in \{1,4\}}p_{\sigma (i)}\times d_{i}}{\sum_{i\in \{1,4\}}d_{i}}\;\times \;0.5, & \mathrm{if}\;d_{1}>d_{4}\\ p_{m}\;\times \;0.5\;+\;\frac{\sum_{i\in \{1,4\}}p_{\sigma (i)}\times d_{5-i}}{\sum_{i\in \{1,4\}}d_{5-i}}\;\times \;0.5, & \mathrm{if}\;d_{1}\leq d_{4} \end{array}\right..$$

Case 3: If $\#\{d_{i}=0\}=1$, there is $p_{\sigma (2)}\;=\;p_{\sigma (3)}\;-\;1$. $p_{e}$ is similarly calculated according to (2).

Case 4: If $\#\{d_{i}=0\}=0$, all neighboring pixels are different. $p_{e}$ is calculated as

(4) $$p_{e}=\left\{\begin{array}{c} min\left(p_{1},p_{3}\right)\;\mathrm{if}\;\;p_{2}\geq max\left(p_{1},p_{3}\right)\\ max\left(p_{1},p_{3}\right)\;\mathrm{if}\;\;p_{2}\leq min\left(p_{1},p_{3}\right)\\ p_{1}+p_{3}-p_{2}\;\mathrm{otherwise} \end{array}\right..$$

After $p_{e}$ is obtained, it is combined with the predefined threshold *t* to activate pixel classification. Before pixel classification is performed, a binary matrix having a size of (m−1)×(n−2) is taken as the location map. For each accessible pixel *p*, if $p_{e}-mod\left(p_{e},T\right)=p-mod\left(p,T\right)$ is fulfilled, it is categorized as Lpxl. Here, $T=2^{\left\lceil log_{2}\left(t\right)\right\rceil }$. Subsequently, the element that locates the corresponding position in the location map is assigned a value of 0. Alternatively, it is classified as NLpxl, and the value of 1 is assigned to its corresponding element of the location map.

**Figure 4:**
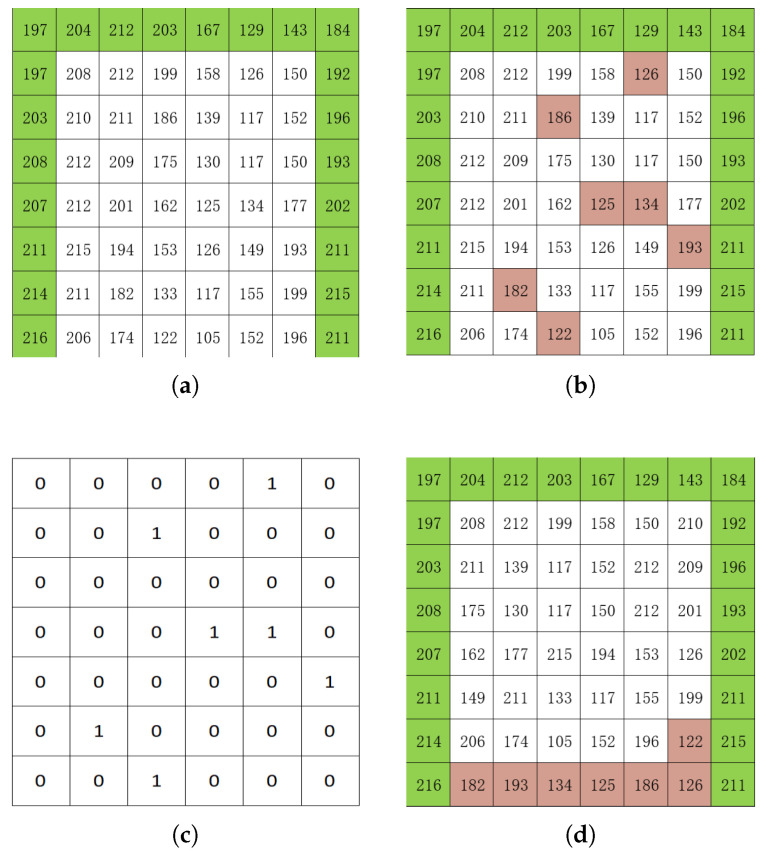
An illustration of pixel classification and image reconstruction while T=64: (**a**) original cover image, (**b**) original cover image with NLpxls marked in pink, (**c**) location map, and (**d**) reconstructed image.

Figure 4 demonstrates the pixel classification workflow with the threshold parameter T=64. Figure 4a displays the original image, where green markers identify candidate pixels for prediction. Figure 4b highlights seven NLpxls detected through our classification protocol, shown in pink. The corresponding location map is presented in Figure 4c, facilitating the achievement of reversible image reconstruction via pixel rearrangement. Figure 4d displays the reconstructed image and reveals the rearrangement rule: Lpxls are ordered first, followed by NLpxls in reverse sequence.

It is notable that, with the assistance of the location map, Figure 4d can be precisely reverted to Figure 4b. Consequently, following the creation of the reconstructed image and location map, the latter is subjected to lossless compression and subsequently embedded into the initial few pixels of the former by substituting $8-\lceil \log_{2}T\rceil$ MSBs. In this paper, the arithmetic compression algorithm is employed to compress the position map. Once the position map has been compressed, its length is recorded and embedded into the initial pixels of the reconstructed image. In the proposed method, the maximum size of the compressed location map can be obtained as

(5) $$MLm=\lceil log_{2}\left(\left(m-1\right)\times \left(n-2\right)\right)\rceil .$$

**Figure 5:**
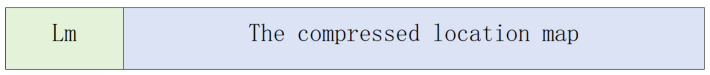
The structure of auxiliary information.

That implies that the first $log_{2}MLm$ bits of the vacated room should be filled with Lm. Accordingly, the first $\left\lceil \frac{log_{2}MLm}{8-\lceil \log_{2}T\rceil }\right\rceil$ pixels of the candidate set within the reconstructed image are occupied by the length of the location map (Lm). Ultimately, the Lm and the compressed location map are organized in the format illustrated in Figure 5 and embedded into the top of the reconstructed image.

The APVCPC exhibits a linear time complexity of O(N), where N is the number of pixels in the candidate set. This complexity arises from processing each pixel a constant number of times: computing its predicted value $p_{e}$ (involving fixed-size neighbor operations and sorting) and then classifying it based on the threshold T. The space complexity is also linear, O(N), mainly due to the storage of the binary location map. Only a small, constant amount of additional working memory is needed for intermediate calculations for each pixel.

### 2.2. Image Encryption

Following the embedding of the location map, image encryption is carried out to avoid the receiver accessing the image content without authentication. For pixel p(i,j) falling into [0, 255], it is expressed in 8 bits by using Equation (6), where ⌊.⌋ represents a rounding down function.

(6) $$p_{k}\left(i,j\right)=\left\lfloor p\left(i,j\right)/2^{k}\right\rfloor mod\;2\;k\in 0,1,2,\cdots ,7$$

Subsequently, leveraging the encryption key in tandem with a stream cipher algorithm, exemplified by RC4 or SEAL, gives rise to the generation of an encryption matrix. This matrix, sized precisely m×n, adheres to the characteristics of a pseudo-random matrix. For each element of the generated matrix (designated as r(i,j)), it is comparably decomposed into 8 bits. Subsequently, encryption is attained by employing Equation (7), where ⊕ represents the XOR operation and the $e_{k}\left(i,j\right)$ stands for the *k*-th encrypted bit.

(7) $$e_{k}\left(i,j\right)=p_{k}\left(i,j\right)⊕r_{k}\left(i,j\right)\;k\in 0,1,2,\cdots ,7$$

Finally, the encrypted pixel is calculated by

(8) $$E\left(i,j\right)=\sum_{k=0}^{7}e_{k}\left(i,j\right)\times 2^{k}.$$

Note that, after the encrypted image is obtained, the Lm should be re-embedded into the initial $\left\lceil \frac{MLm}{8-\lceil \log_{2}T\rceil }\right\rceil$ pixels within the candidate set.

### 2.3. Data Hiding

In this step, some confidential data, including access control information, user details, or copyright information, are embedded by the data hider. To ensure the confidentiality and security of this sensitive information, the data are encrypted using the data-hiding key prior to the initiation of the data hiding process. Subsequently, the Lm is extracted from the first $\left\lceil \frac{log_{2}MLm}{8-\lceil \log_{2}T\rceil }\right\rceil$ pixels within the candidate set of the received image, thereby determining the starting position of the available embedding space (i.e., the “vacated room” referenced in the original text). Then, by replacing $8-\lceil log_{2}T\rceil$ MSBs, the encrypted confidential data are concealed in Lpxls except for the initial few ones. It should be noted that once all confidential bits have been embedded, an end mark, such as 16 consecutive “0”s, should be embedded.

### 2.4. Data Extraction and Image Recovery

The proposed method enables the accomplishment of separable execution regarding image recovery and data extraction. Based on their access to the data-hiding key and decryption key, three categories of receivers are identified in the proposed method.

Case 1: Receivers having the data-hiding key but lacking the decryption key.

For the recipients of this category, the image content is inaccessible. However, the embedded data can be retrieved without any degradation in fidelity. In specific terms, the $8-\lceil log_{2}T\rceil$ MSBs of the initial $\left\lceil \frac{log_{2}MLm}{8-\lceil \log_{2}T\rceil }\right\rceil$ pixels are first extracted; this step serves to acquire the starting position of the confidential data, which is prerequired for secret data extraction. Subsequently, the same number of MSBs are sequentially extracted from the remaining Lpxls, commencing from the identified start point and continuing until a predefined end marker (e.g., 16 consecutive zeros) is detected. Finally, the complete set of extracted bits is decrypted using the data-hiding key, thereby perfectly reconstructing the original confidential data. Moreover, the data recovery exhibits O(n) time complexity, where N is the number of embedded secret bits, dominated by the sequential MSB extraction from Lpxls and the subsequent decryption, both linear operations. The initial position retrieval requires only constant time. Space complexity is also O(n), primarily for storing the extracted bitstream before decryption, with O(1) additional working memory.

Case 2: Receivers possessing decryption key but lacking the data-hiding key.

**Figure 6:**
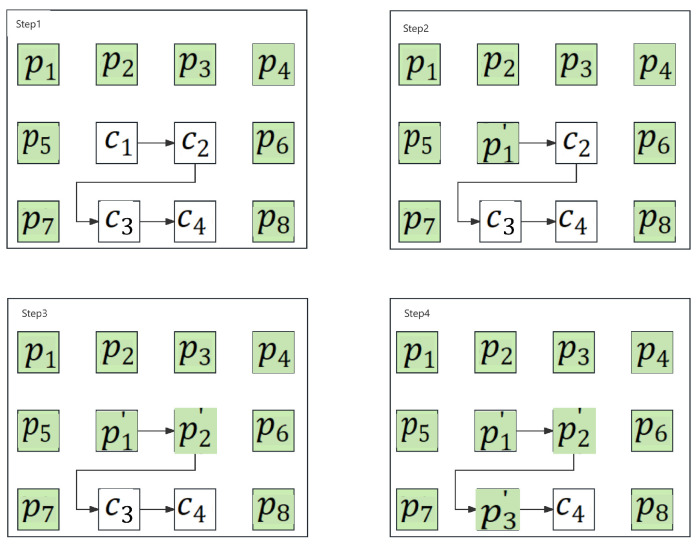
Demonstration of pixel restoration.

In this scenario, although these receivers cannot access the confidential data, they are still able to obtain the content of the original image. The recovery process, which shares its initial step with Case 1, begins by extracting the auxiliary data (Lm) from the first $8-\lceil log_{2}T\rceil$ MSBs of the initial $\left\lceil \frac{log_{2}MLm}{8-\lceil \log_{2}T\rceil }\right\rceil$ pixels within the candidate set. Subsequently, the received image is decrypted by employing the decryption key; following this decryption process, the compressed location map is further extracted on the basis of the retrieved Lm. Thereafter, the original location map can be effectively retrieved through decompression. On this basis, all Lpxls and NLpxls are distinguished and repositioned to their original positions in line with the retrieved location map. Subsequently, all pixels within the candidate set are restored in a raster scanning sequence, as illustrated in Figure 6. Here, the pixels within the prediction set are marked in green, while those in the candidate set are marked in white. As it is shown in Step 1, the candidate pixel $c_{1}$ is retrieved initially. Specifically, its predicted value $p_{e1}$ is calculated with the assistance of its neighboring pixels $p_{1}$, $p_{2}$, $p_{3}$ and $p_{5}$, adopting the same methodology as that employed in pixel classification. Upon obtaining the predicted value $p_{e1}$, the original pixel is restored through the application of Equation (9), where $p_{1}^{\prime }$ denotes the restored value of the pixel $c_{1}$. Following this restoration, the recovered value $p_{1}^{\prime }$ becomes one of the neighboring pixels of $c_{2}$. With the support of the pixels $p_{1}^{\prime }$, $p_{2}$, $p_{3}$, and $p_{4}$, $c_{2}$ is then restored to its original state, denoted as $p_{2}^{\prime }$. Thereafter, as illustrated in Figure 6, the candidate pixels $c_{3}$ and $c_{4}$ are restored in the same manner as $c_{1}$ and $c_{2}$. Upon completion of all these restoration steps, the original image is successfully retrieved.

Notably, the restoration involves linear traversal of candidate pixels and temporary storage of their restored values without redundant operations, resulting in a time complexity and a space complexity of O(N), respectively (N is the total number of candidate pixels)

(9) $$p_{1}^{\prime }=p_{e1}\;-\;mod\left(p_{e1},T\right)\;+\;mod\left(c_{1},T\right)$$

Case 3: Receivers are entitled to access all keys.

In this case, the encrypted confidential data are first extracted from the received image. Subsequently, with the assistance of data-hiding key, receivers in this category can access the confidential data. After that, the original image is perfectly retrieved by utilizing the decryption key. Moreover, the time complexity and the space complexity of this scenario are the sum of that in Case 1 and Case 2.

## 3. Experimental Results and Analyses

In this section, a series of experiments, including image recovery, data extraction, performance analysis, and comparison with existing methodologies, are conducted to evaluate the performance of the proposed method. To rigorously evaluate the performance of the proposed reversible data hiding in encrypted images (RDHEI) scheme, this study employs a diverse set of eight standard grayscale test images. These include the widely adopted “Lenna” image—selected for its balanced composition of smooth regions (e.g., facial skin and background) and highly textured areas (e.g., hair and hat)—which makes it particularly suitable for comprehensive assessment of image processing algorithms. Complementing “Lenna”, the testing set comprises the relatively smooth “Peppers” image, moderately textured images (“Barbara”, “F16”, and “Boat”), and highly textured images (“Lake” and “Tank”). This deliberate selection ensures systematic evaluation across varying structural complexities and texture densities. The aforementioned eight images have dimensions of 512×512. All programs were coded in MATLAB R2016a. The experimental configuration utilized a PC furnished with a 64-bit Windows 11, 16 GB of RAM, and an Intel Core CPU operating at a speed of 1.8 GHz.

### 3.1. Image Recovery and Data Extraction

**Figure 7:**
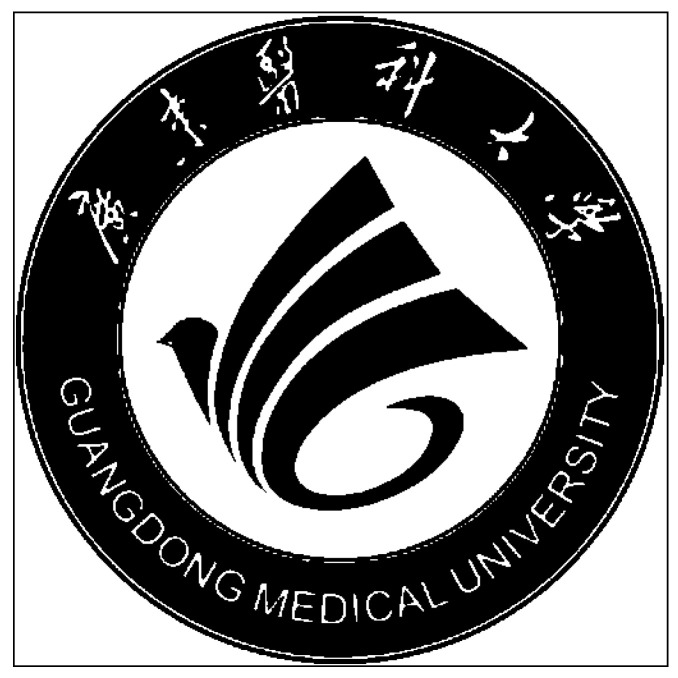
Secret image with a size of 512×512.

**Figure 8:**
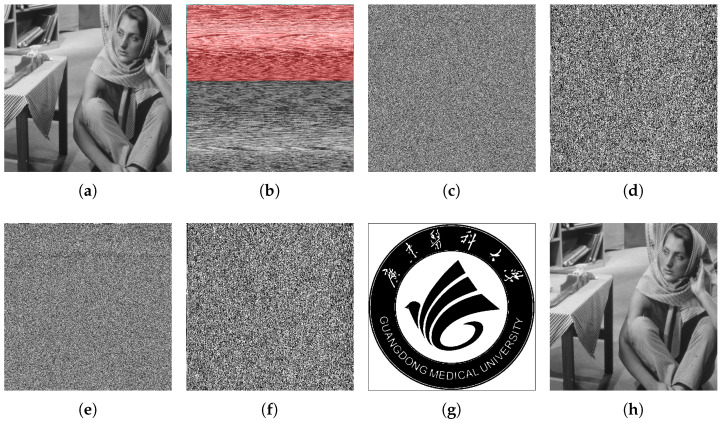
Images of several key phases of the proposed method are presented as follows: (**a**) the original cover image, which serves as the cover image; (**b**) the reconstructed image, in which the Lpxls are marked in gray; (**c**) the encrypted image, which resembles a noisy image; (**d**) the encrypted confidential data, containing none of the content of the secret image; (**e**) the marked encrypted image, which carries the secret image; (**f**) the extracted data without decryption; (**g**) the extracted data after decryption, which is the same as the original secret image; (**h**) and the recovered image, which is the same as the original image.

In this experiment, as presented in Figure 7, the confidential data are in the form of a binary image having a size of 512×512. The standard test image “Lenna” is selected as the cover image. It is of utmost importance to note that, for the “Lenna” image to be able to carry the confidential data, the embedding rate must reach 1 bpp. Figure 8 presents the results of several key stages of the proposed method, with the encryption key set as “123456” and T = 8. Figure 8b shows the reconstructed image, where Lpxls are highlighted in red. The observation demonstrates that more than half of the cover image consists of Lpxls when T = 8, suggesting that a high capacity can be attained. Figure 8c displays the encrypted image, and it is impossible to capture any contents of the original image. Therefore, the image contents have been effectively safeguarded through the application of image encryption. The encrypted result of the confidential data and its embedded output are presented in Figure 8d,e. The directly extracted data, as illustrated in Figure 8f, indicate that the confidential content remains undetectable to receivers without data-hiding key. The extracted result is shown in Figure 8f, and its decrypted result is presented in Figure 8g. As can be seen, the decrypted result perfectly aligns with Figure 7. This indicates that the extraction of hidden data is flawless, thereby guaranteeing its reversibility. Similarly, the recovered result of the received image is presented in Figure 8h. As can be seen, the recovered result is precisely identical to Figure 8a, which depicts the original image. This indicates that the reversibility of the cover image is achieved in the proposed method.

According to the discussion mentioned above, the reversibility of the RDHEI is accomplished by the proposed method. Simultaneously, the contents of confidential data and the original image are effectively protected.

### 3.2. Performance Analysis

**Figure 9:**
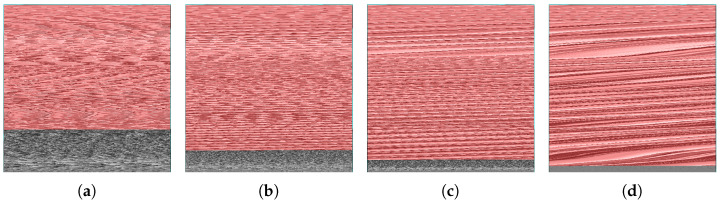
Reconstructed results of Lenna with different threshold *T*: (**a**) *T* is 16, (**b**) *T* is 32, (**c**) *T* is 64, and (**d**) *T* is 128.

It is obvious that the quantity of Lpxls varies in accordance with the parameter *T* and the cover image. To verify the influence of these two factors on the performance of the proposed method. All the aforementioned test images were utilized to assess the performance of the proposed method. Figure 9 presents the reconstructed results of “Lenna” for T∈{16,32,64,128}, with Lpxls highlighted in red. It can be observed that the red area expands as *T* increases. This indicates that a larger *T* results in a greater quantity of Lpxls. The embedding capacity, in the proposed method, is obtained by Equation (10), where *N* and Lm represent the quantity of Lpxls and the length of the compressed location map, respectively.

**Figure 10:**
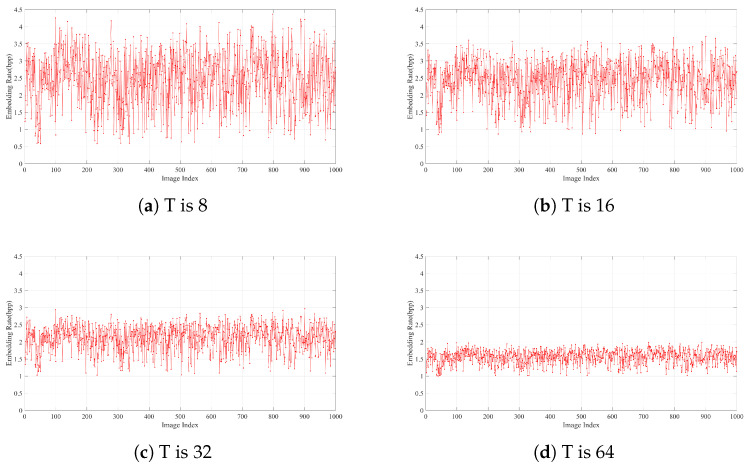
Performance evaluation using different values of parameter “T” on an additional set of 1000 images from the BOW-2 image database.

For the purpose of more effectively evaluating the performance of the proposed method, an additional set of 1000 images from the BOW-2 database (https://data.mendeley.com/datasets/kb3ngxfmjw/1, accessed on 2 June 2023) is utilized as test images. Figure 10a presents the test results when “8” is set as *T*. As depicted, the embedding rates of the majority of test images exceed 4 bpp. In contrast, only a small number of images have embedding rates below 1 bpp. The occurrence of embedding rates lower than 1 bpp becomes increasingly rare with T=16, as illustrated in Figure 10b. However, for T=32 or T=64, the embedding rate consistently exceeds 1 bpp, as demonstrated in Figure 10c,d. These experimental results indicate that although a larger *T* can increase the embedding rate for some test images, Figure 10 reveals that it actually leads to a decrease in the embedding rate for the majority of test images. A detailed quantitative summary of the results on the 1000 BOW-2 images is provided in Table 3. The data show that the highest embedding capacity (EC) is attained at T=8, where the average embedding rate (ER) reaches 2.48 bpp—both measures surpassing those achieved with other *T* values. This observation initially indicates that T = 8 may be the optimal threshold parameter for the majority of cover images. However, Table 3 also highlights that the lowest recorded ER at T=8 is 0.58 bpp, which represents the poorest minimum performance among all tested thresholds. In contrast, the best minimum EC is observed at T=32 (1.02 bpp), closely followed by T=64 (1.01 bpp). This implies that, for certain specific images—presumably those with less favorable local pixel distributions—adopting a higher *T* value (32 or 64) can yield a more reliable minimum embedding capacity. Furthermore, the proposed method maintains an average embedding rate above 2 bpp for T∈8,16,32, confirming its ability to deliver high embedding capacity across multiple parameter settings. Collectively, these comprehensive experiments—spanning multiple threshold configurations and a large, diverse test dataset—robustly validate the conclusion that the proposed method achieves a substantially high embedding capacity, with its performance characteristics adaptable to different image types through appropriate adjustment of the threshold *T*.

(10) $$EC=N\times (8-log_{2}T)-Lm.$$

For a better comprehension of the relationship between the performance and the parameter *T*, Table 4 enumerates the performance of all test images for different *T*. It is observable that the quantity of Lpxls typically ascends as *T* increases. Nevertheless, the $8-\lceil log_{2}T\rceil$ MSBs of Lpxls are employed for data hiding. Hence, it cannot be inferred that the parameter *T* and the embedding capacity are in a positive correlation.

Therefore, the proposed method is extremely appropriate for the practical applications of RDHEI.

### 3.3. Comparison with Several Existing Methods

**Figure 11:**
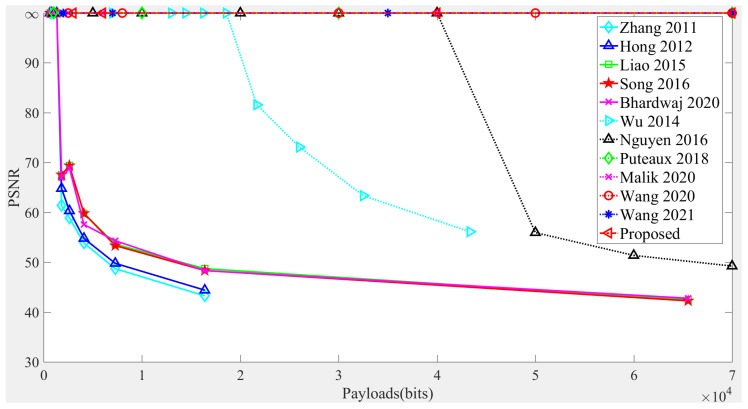
PSNR comparison of several previous methods and the proposed method on the image Lenna for different payloads [7,8,9,10,11,12,26,31,33,34,35].

Fist of all, the standard image “Lenna” is selected to test the Peak Signal-to-Noise Ratio (PSNR) of the proposed method and several state-of-the-art RDHEI methods. Here, PSNR is employed as a quantitative measure to evaluate the disparity between the recovered cover images and the original ones. Subsequently, the embedding capacity is utilized to compare their respective performances. Figure 11 illustrates the PSNR comparison at different payload levels, with Lenna being used as the cover image. It is observed that the PSNRs of methods [7,8,9,10,11,12,26] cannot remain at +∞ for all payloads, suggesting a loss of reversibility. In contrast, for the given payloads, the methods in [31,33,34,35] and the proposed method consistently yield a PSNR of +∞. This suggests that the reversibility is achieved by methods [31,33,34,35] and the proposed method.

Table 5 presents a detailed delineation of the embedding capacities obtained by the proposed method and several prior ones on the eight aforementioned test images. As Table 5 illustrates, the proposed method exhibits a markedly superior embedding capacity over previous methods, with a prominent margin in performance. The maximum capacity of the image “Lenna” achieved by the previous methods reaches 477,282 bits. However, with a capacity of 564,116 bits, the proposed method achieves a remarkable increase over previous approaches. For these eight test images, the proposed method achieves an average capacity of 500,710 bits, compared with an average capacity ranging from 922 to 430,797 bits for previous methods. Evidently, the proposed method has achieved a significant superiority in capacity. Based on the foregoing discussion, it can be rationally postulated that the proposed method distinctly exhibits an augmented and preponderant suitability for practical RDHEI applications.

### 3.4. Security Analysis

Attackers can be categorized into three groups based on the keys they possess. The first group encompasses those solely in possession of the data-hiding key, with the objective of attaining access to the image content. The secondary group comprises those who only possess the decryption key and harbor the intention of extracting the confidential data. The tertiary group consists of those lacking both keys but endeavoring to access both the image content and the confidential data.

The security scrutiny pertinent to each attacker category is elucidated hereinafter, commensurate with the attributes of their motives and competencies.

Case 1: Attackers possessing only the data-hiding key.

With the application of the data-hiding key, the extraction and decryption of the confidential data can be achieved without any detriment in this given situation. He can obtain the secret data as shown in Figure 8g. As we can see, the content of secret data is readable for this kind of attackers. However, as visualized in Figure 8c, the encrypted image can be obtained in this scenario. However, due to the non-availability of the decryption key, the image decryption becomes impracticable. Thus, the safety and wholeness of the original cover image stay unaltered and intact.

Case 2: Attackers possessing only decryption key.

In this scenario, an attacker can successfully extract the auxiliary location map (Lm) from the leading pixels of the candidate set. Consequently, the starting position of the secret data payload can be determined. Following this, the encrypted secret data themselves can be retrieved, as visually outlined in Figure 8d. However, the absence of the data-hiding key prevents the attacker from performing the final decryption operation on the extracted ciphertext. As a result, both the integrity and confidentiality of the embedded information remain fully preserved against this type of adversarial access.

Case 3: Attackers without any key.

In this scenario, attackers have access to both the encrypted secret data (Figure 8d) and the encrypted image (Figure 10c), facilitated by the Lm. Critically, however, the absence of both the decryption key and the data-hiding key renders them incapable of executing either decryption operation. Consequently, the privacy of the original image content and the security (i.e., integrity and confidentiality) of the embedded confidential data remain entirely preserved against such attackers.

As outlined above, the proposed method effectively guarantees the security of confidential data and protects the privacy of image owners.

## 4. Conclusions

This paper presents an advanced RDHEI framework, termed APVCPC, designed to enhance embedding capacity while concurrently ensuring robust security and bit-to-bit reversibility for visual sensing assets. The core innovation lies in an adaptive prediction engine that dynamically computes pixel estimates based on local contextual complexity, significantly improving the precision of spatial redundancy utilization. By leveraging the alignment between most significant bits (MSBs) and these adaptive predictions, the proposed pixel classification paradigm effectively identifies optimal regions for data embedding. Experimental evaluations across diverse image datasets demonstrate that our method consistently outperforms state-of-the-art approaches in terms of embedding rate and reconstruction fidelity. Furthermore, the architecture supports fully separable operations for data extraction and image recovery, providing flexible access control for cloud-based storage. Nevertheless, the present work primarily focuses on maximizing embedding capacity, with less emphasis on a detailed analysis of the algorithm’s space and time complexity—specifically, its execution speed and memory consumption during operation. Additionally, the robustness of the scheme against potential corruption of the embedded auxiliary data (e.g., the location map) has not been addressed. These identified aspects constitute promising directions for future research. Subsequent work will therefore concentrate on developing algorithms that holistically balance embedding capacity, computational efficiency, and storage overhead. Concurrently, investigations will be undertaken to enhance the algorithm’s robustness, ensuring reliable data recovery even under conditions of partial auxiliary information loss. Given its high embedding capacity, the proposed scheme offers a promising solution for safeguarding information security in remote monitoring systems.

## Figures and Tables

**Table 1:** The symbols used in this paper and their meanings.

Symbol	Exact Meaning	Symbol	Exact Meaning
Lpxl	Loadable pixel, used to carry extra data	Nlpxl	Non-loadable pixel, cannot be modified
*p*	Candidate pixel	$p_{e}$	Predicted value of the candidate pixel
$p_{1}$	Adjacent pixel 1 of the candidate pixel	$p_{2}$	Adjacent pixel 2 of the candidate pixel
$p_{3}$	Adjacent pixel 3 of the candidate pixel	$p_{4}$	Adjacent pixel 4 of the candidate pixel
$p_{\sigma 1}$	Minimum value among adjacent pixels	$p_{\sigma 2}$	Second minimum value among adjacent pixels
$p_{\sigma 3}$	Second maximum value among adjacent pixels	$p_{\sigma 4}$	Maximum value among adjacent pixels
$p_{m}$	Average of the second minimum and second maximum values	$d_{i}$	Absolute difference between the *i*-th minimum value and $p_{m}$
*t*	A predefined threshold	*T*	Normalized threshold processed by $2^{\left\lceil \log_{2}^{\left(t\right)}\right\rceil }$
MLm	Maximum length of the location map	Lm	Length of the compressed location map
EC	Embedding capacity	bpp	Bits per pixel, extra data bits per pixel
$e_{k}\left(i,j\right)$	The *k*-th bit of the pixel at row *i*, column *j* in the encrypted image	$r_{k}\left(i,j\right)$	The *k*-th bit of the pixel at row *i*, column *j* in the random matrix
E(i,j)	The pixel at row *i*, column *j* in the encrypted image		

**Table 2:** The function symbols involved in this article and their functions.

Function	Role	Function	Role
round()	Rounding operation	#{}	Set of elements satisfying a condition
max()	Compute the maximum value	min()	Compute the minimum value
mod(a,b)	Compute *a* modulo *b*	$\log_{b}a$	Logarithm of *a* to the base *b*
⌈·⌉	Ceiling function	⌊·⌋	Floor function

**Table 3:** Performance summary of the proposed method on extra 1000 images of the BOW-2 database with various *T*.

		Highest	Lowest	Average
T = 8	EC	1,145,070	152,706	646,179
	bpp	4.37	0.58	2.48
T = 16	EC	973,393	221,877	639,008
	bpp	3.71	0.85	2.44
T = 32	EC	776,992	268,157	556,755
	bpp	2.96	1.02	2.12
T = 64	EC	520,257	262,626	407,840
	bpp	1.99	1.01	1.56

**Table 4:** Performance of the proposed method with various *T*.

		Barbara	Boat	F16	House	Lake	Lenna	Peppers	Tank
T = 4	N	75,082	63,607	127,337	110,500	61,234	98,155	71,953	69,184
	Lm	225,774	208,961	260,531	256,269	205,006	249,066	221,557	217,599
	EC	224,718	172,681	503,491	406,731	162,398	339,864	210,161	197,505
	bpp	0.86	0.66	1.92	1.55	0.62	1.3	0.8	0.75
T = 8	N	118,533	111,249	172,084	151,119	102,231	151,286	123,729	119,017
	Lm	259,093	256,594	240,958	255,812	251,834	255,734	260,150	259,216
	EC	333,572	299,651	619,462	499,783	259,321	500,696	358,495	335,869
	bpp	1.27	1.14	2.36	1.91	0.99	1.91	1.37	1.28
T = 16	N	159,467	166,276	207,583	191,105	157,619	194,332	177,648	176,822
	Lm	251,133	246,116	189,956	218,069	252,307	213,212	235,237	236,135
	EC	386,735	418,988	640,376	546,351	378,169	564,116	475,355	471,153
	bpp	1.48	1.6	2.44	2.08	1.44	2.15	1.81	1.8
T = 32	N	193,835	213,127	234,103	220,725	204,545	226,682	215,113	222,972
	Lm	213,979	178,501	123,652	160,922	195,783	145,427	174,125	155,265
	EC	367,526	460,880	578,657	501,253	417,852	534,619	471,214	513,651
	bpp	1.4	1.76	2.21	1.91	1.59	2.04	1.8	1.96
T = 64	N	224,889	241,569	243,780	239,355	229,812	241,393	241,284	240,352
	Lm	150,262	98,335	90,023	106,256	136,604	98,979	99,376	102,736
	EC	299,516	384,803	397,537	372,454	323,020	383,807	383,192	377,968
	bpp	1.14	1.47	1.52	1.42	1.23	1.46	1.46	1.44
T = 128	N	241,641	248,915	254,979	249,972	252,609	251,000	253,018	243,318
	Lm	98,071	68,877	39,208	64,139	51,593	59,379	49,540	91,796
	EC	143,570	180,038	215,771	185,833	201,016	191,621	203,478	151,522
	bpp	0.55	0.69	0.82	0.71	0.77	0.73	0.78	0.58

**Table 5:** Capacity comparison with several previous methods (unit: bits).

Image	[7]	[8]	[9]	[10]	[11]	[12]	[26]	[33]	[31]	[34]	[35]	Proposed
Barbara	225	225	196	225	436	43,350	34,342	253,576	160,693	164,815	334,255	386,735
Boat	1024	1764	1296	1024	1875	32,512	27,262	259,864	191,112	197,305	377,348	460,880
F16	196	196	196	196	1250	43,350	73,139	260,208	192,135	303,592	591,496	640,736
House	81	144	64	81	162	26,010	44,009	260,214	190,402	207,200	467,542	546,351
Lake	1024	1296	784	1024	1323	26,010	29,750	259,896	192,244	183,805	363,709	417,852
Lenna	1764	1296	1764	1296	1875	26,010	41,157	258,760	184,744	229,350	477,282	564,116
Peppers	1296	2601	1024	1296	1875	32,512	41,419	260,304	192,172	214,325	452,842	475,355
Tank	1764	1764	1764	1764	2352	32,512	21,510	260,196	155,161	220,415	381,906	513,651
Average	922	940	688	863	1394	32,783	36,385	259,127	182,333	215,101	430,797	500,710

## Data Availability

The original contributions presented in this study are included in the article. Further inquiries can be directed to the corresponding author.
